# Globalization of pediatric research: pharmacological RCTs in Latin America

**DOI:** 10.1186/s13052-019-0622-1

**Published:** 2019-03-04

**Authors:** Federica Arienti, Claudia Pansieri, Chiara Pandolfini, Andrea Biondi, Maurizio Bonati

**Affiliations:** 1 Laboratory for Mother and Child Health, Department of Public Health, IRCCS - Istituto di RicercheFarmacologiche “Mario Negri”, Via Giuseppe la Masa 19, 20156 Milan, Italy; 2 Department of Pediatrics, Hospital S. Gerardo/Fondazione MBBM, University of Milano-Bicocca, Monza, Italy

**Keywords:** Globalization, Developing countries, Latin America, Randomized clinical trials

## Abstract

Globalization caused a shift in trial locations towards low-middle income countries, raising ethical concerns. These include the risk that conditions primarily affecting children in these countries will be neglected in favor of those affecting developed countries. We analyzed 253 published and 69 ongoing pharmacological RCTs performed in Latin America between 2000 and 2015 involving exclusively children. While over 50% of the previously highly investigated diseases were no longer priorities, other diseases acquired greater attention in recent years. Brazil and Mexico resulted as the most active countries. A large gap remains between the real needs of children in these countries and scientific research.

The number of randomized controlled trials (RCTs) in children, limited in the past due to ethical, physiological, pharmacometric challenges [1] and to low profitability, [2] has grown following several legislative initiatives, albeit with continuing challenges [3] and considerable heterogeneity across geographical regions. [4] In this context, we performed a literature review in Medline to determine the number of pediatric clinical trials conducted in Latin America and the Caribbean (LAC) in the last 16 years (01/01/2000–31/12/2015 period) to understand if the therapeutic areas studied in these low-middle income countries (LMICs) were pertinent to local health care needs. We compared these results with ongoing studies, and therefore with the future direction of research, obtained via a scoping review of ClinicalTrials.gov. Only RCTs classified as “Drug” were retained and analyzed further. Furtermore, we chose the three most prevalent diseases, and compared them with the conditions causing the greatest pediatric burden of disease in LAC [5].

253 of 1254 (21%) published and 69 ongoing pharmacological RCTs resulted pertinent and were analyzed. The majority of these (60%) were conducted in Brazil and Mexico. Diseases of the respiratory system, followed by certain infectious and parasitic diseases were the target of the majority of both published (33%) and ongoing (41%) RCTs.Over 50% of the previously highly investigated diseases, such as the Symptoms, Signs and Abnormal Clinical and Laboratory Findings not elsewhere classified (SSACLF), and certain conditions originating in the perinatal period, were no longer priorities.

The three most prevalent diseases, asthma, pneumonia, and giardiasis, had 21, 9, and 8 published RCTs, respectively. Salbutamol was the most used drug in the asthma trials (5/18; 28%), while, among the systemic antibiotics, amoxicillin was the most studied in bacterial pneumonia treatment (3/5; 60%). Mebendazole was the most used drug for giardiasis (3/7; 43%). Of the ongoing studies, 9 concerned asthma, 5 pneumonia, and none addressed giardiasis. The majority of the drugs being studied for asthma were inhaled corticosteroids such as fluticasone and beclomethasone. Against viral pneumonia, 4 RCTs concerned new generation monoclonal antibodies and one trial studied an old antiviral drug, oseltamivir. None of the ongoing trials address bacterial pneumonia or giardiasis.

In the last 16 years, the most studied drugs in the treatment of asthma were Short Acting β-Adrenergic and Long Acting β-Adrenergic (LABA), the two classes of β-Adrenergic drugs recommended by the The Global Initiative for Asthma 2015 guidelines. In the ongoing trials, different dosages of LABA and inhaled corticosteroids were being analyzed in safety and efficacy studies. Magnesium sulphate was also being studied in one Mexican, national level, ongoing efficacy trial. Only 3/9 ongoing trials on asthma were placebo-controlled.

Regarding pneumonia, the published studies mostly concerned the treatment of Community-Acquired Pneumonia with amoxicillin against *S. pneumoniae* or azithromycin against *M. Pneumoniae*, the two first line therapies recommended by the Infectious Diseases Society of America 2013 guidelines, while the ongoing research is focusing exclusively on the treatment of pneumonia caused by Respiratory Syncytial Virus with new generation monoclonal antibodies. All these RCTs were placebo-controlled, while the only pharmacokinetic trial concerned oseltamivir, the antiviral drug already used as a first line therapy for influenza. Finally, giardiasis has been treated with the most common anti-parasitic drugs in the last 16 years, as recommended by the World Gastroenterology Organization 2012 guidelines, and no clinical trial is currently underway on this disease. See Table 1 for details.

**Table 1:** Comparison with the guidelines of the drugs studied in the published articles and the ongoing RCTs for the three prevalent diseases

Condition Investigated	Published (2000–2015)	Guidelines	Treatment	Ongoing (2015)
**Asthma**	Salbutamol (SABA)	GINA (The Global Initiative for Asthma) 2015	Step 1: SABA	Fluticasone/Salmeterol (ICS + LABA)
	Formoterol (LABA)		Step 2: low dose ICS + SABA	Beclometasone dipropionate (ICS)
	Salmeterol (LABA)		Step 3: low dose ICS/LABA + SABA	Budesonide/Formoterol (ICS + LABA)
	Montelukast (LTRA)		Step 4: medium dose ICS/LABA + SABA	Dexamethasone/Epinephrine (ICS)
	Fluticasone (ICS)		Step 5: add-on treatment (omalizumab or tiotropium)	Salbutamol (SABA)
	Budesonide (ICS)			Magnesium Sulphate
	Aminophylline			Lebrikizumab
	Triamcinolone			
**CAP**	Amoxicillin	IDSA (The Infectious Diseases Society of America) 2013	**Outpatients**	
	Azithromycin		Amoxicillin	
	Chloramphenicol		Macrolide antibiotic	
			**Inpatients**	
			First line: Ampicillin or Penicillin G	
			Second line: Ceftriaxone or Cefotaxime	
			Macrolide + β-lactam antibiotic	
			Vancomycin or Clindamycin + β-lactam antibiotic	
**Viral Pneumonia**	Palivizumab		**Influenza**	Oseltamivir
	Motavizumab		Amantadine or Rimantadine	MEDI8897
			Oseltamivir or Zanamivir	ALS-008176
			**RSV**	REGN2222
			Palivizumab	JNJ-53718678
			Ribavirin	
**Giardiasis**	Mebendazole	WGO (The World Gastroenterology Organization) 2012	First line: Metronidazole	
	Albendazole		Second line: Tinidazole or Ornidazole	
	Nitazoxanide			
	Chloroquine			
	Tinidazole			

The number of published pediatric trials conducted in LAC has increased over the last 16 years, although this area’s low investment in research remains a concern, [6] and may be due to the increased complexity of conducting trials in children in LMICs [7].

Many trials designed to improve therapies aimed at the US population enroll patients in a variety of countries. Up to one third of published pediatric clinical trials involve patients in LMICs [8]. This globalization of pediatric research has significant scientific advantages, including evaluating safety and efficacy in more heterogeneous populations and increasing the opportunity to impact on child health on a global scale, [8] but it also raises scientific and ethical concerns, including whether the research is relevant to the needs of the local populations, the scientific validity of extrapolating results from different patient populations, and the availability of health-related resources and therapies once trials end [9]. As in our findings, the therapeutic areas covered by published pediatric trials conducted in LAC seem appropriate given the burden of disease of the related diseases in the region. Despite the focus on respiratory, anti-infective and antiparasitic drugs, several other important research areaslack in public health intervention, such as injuries, congenital diseases, and perinatal conditions. Our findings are in line with recent evidence showing that in LMICs there has been a shift towards noncommunicable diseases (NCDs) [8] that are rightly being given increased attention. The rise of transitional, economy driven research, will place greater attention on NCDs, such as type 2 diabetes, increasing the risk that conditions primarily affecting children in the poorest regions of the world will be neglected [10].

The increasing attention on asthma treatment is justified by the high prevalence of this disease. The published research, such as the montelukast studies, seems to reflect the needs of children in high income countries, since this kind of drug is not easily accessible in LAC due to issues on price and distribution. Furthermore, the ongoing research on drugs such as beclometasone dipropionate and fluticasone/salmeterol, which are already contemplated in the practice guidelines, adds little new knowledge to the treatment of this growing disease. On the other hand, magnesium sulphate, addressed by an ongoing Mexican efficacy trial, really reflects current LAC pediatric research needs.

Regarding pneumonia, attention shifted from treatment, which is still reflected in the guidelines, to preventive strategies with new generation drugs,such as monoclonal antibodies,once again, not in favor of the pediatric patients of these countries.

*Giardia* is still a major cause of mortality and morbidity in this region but despite this, no new therapeutic strategy is currently being studied, and research focuses on preventive therapies that, however, are of poor economic interest for the rest of the world.

## Conclusions

Much remains to be done to respond to the real needs of the pediatric population in these countries, as seen with the lack of ongoing trials on a disease with a large impact on mortality, such as *Giardia*. Scientific research addressing the many different pediatric infectious diseases should be made a worldwide priority and should involve collaborative efforts between countries, investigators, policy makers, and industry.

## Abbreviations

ICS: Inhaled corticosteroids
IDSA: The Infectious Diseases Society of America
LABA: Long Acting β-Adrenergic
LAC: Latin America and the Caribbean
LMICs: low-middle income countries
NCDs: noncommunicable diseases
RCT: randomized controlled trials
SABA: Short Acting β-Adrenergic
SSACLF: Symptoms, Signs and Abnormal Clinical and Laboratory Findings not elsewhere classified
